# Supplementary material for: Widespread discrepancy in Nnt genotypes and genetic backgrounds complicates granzyme A and other knockout mouse studies
Source: eLife. 2022 Feb 4;11:e70207. doi: 10.7554/eLife.70207 (PMC8816380; doi:10.7554/eLife.70207)
Supplement: Supplementary file 1. — Compilation of studies employing Gzma-/- or Gzma-/- Gzmb-/- double KO mice that reveal a phenotype or do not show a phenotype. [file elife-70207-supp1.docx]

**Supplementary File 1. Studies using *Gzma-/-* mice.**

| **Refs** | **Mice** | **Studies using *Gzma-/-* mice showing a phenotype** | **Year** |
| --- | --- | --- | --- |
| ^1^ | *Gzma-/-* | Gzma is critical for recovery of mice from infection with ectromelia | 1996 |
| ^2^ | *Gzma-/-* | Lack of Gzma increases the virulence of cowpox virus | 1999 |
| ^3^ | *Gzma×b-/-* | Gzma initiates an alternative pathway for granule-mediated apoptosis | 1999 |
| ^4^ | *Gzma-/-* | Increased virus load in spinal ganglia of *Gzma-/-* mice | 2000 |
| ^5^ | *Gzma-/-* mice, *Gzmb-/-* mice, *Gzma×b-/-* | Gzma and B and the Gzma substrate, caspase 3, are important for regulating latent HV68 infection | 2004 |
| ^6^ | *Gzma-/-*, *Gzmb-/-* | *Gzma-/-* mice resist lipopolysaccharide-induced toxicity | 2008 |
| ^7^ | *Gzma-/-* | Gzma as critical effector molecule of human Treg function for gastrointestinal immune response in an experimental GvHD model. | 2015 |
| ^8^ | *Gzma-/-* | 1 *Gzma-/-* mice showed a better survival and lower bacterial counts in BALF and distant body sites. 2. Gzma enhances the early inflammatory response in the lung during pneumococcal pneumonia | 2016 |
| ^9^ | *Gzma-/-* mice, *Gzmb-/-* mice, *Gzma×b-/-* | Deficiency of Gzma and/or Gzma associated with increased bacterial loads | 2017 |
| ^10^ | *Gzma-/-* | NOD Mice lacking Gzma develop increased autoimmune diabetes and increased expression of type I IFN–regulated genes | 2017 |
| ^11^ | *Gzma-/-* | Gzma contributes to inflammatory arthritis in mice through stimulation of osteoclastogenesis | 2017 |
| ^12^ | *Gzma-/-* | Gzma–producing T helper cells are critical for acute graft-versus-host disease | 2020 |
| ^13^ | *Gzma-/-* | Extracellular Gzma Promotes Colorectal Cancer Development by Enhancing Gut Inflammation | 2020 |
| ^14^ | *Gzma-/-*, *Gzmb-/-*,  *Gzma×b-/-* | Gzma induces a novel death with writhing morphology, 'athetosis' | 2013 |
| ^15^ | *Gzma-/-*, *Gzmb-/-*, GzmaxB-/- | infection-related pathology, but not bacterial clearance, appears to require Gzma | 2014 |
|  | | | |
| **Studies using *Gzma-/-* ; *Gzmb-/-* double KO mice showing a phenotype** | | | |
| ^16^ | *Gzma-/-*, *Gzmb-/-*, *Gzma×b-/-* | Lack of Gzma or Gzma renders mice 10 to 100-fold more susceptible to Primary Ect Virus Infection; Gzma and Gzma are effector molecules in granule exocytosis-mediated host defense | 1999 |
| ^17^ | *Gzma-/-*, *Gzmb-/-*, *Gzma×b-/-* | Gzma/B contribute to viral elimination in salivary glands | 2000 |
| ^18^ | *Gzma-/-*, *Gzmb-/-*, *Gzma×b-/-* | Gzma and Gzma are required for NK cell-mediated tumor control in vivo | 2002 |
| ^19^ | *Gzma-/-*, *Gzmb-/-*, *Gzma×b-/-* | Gzma and Gzma partly regulate local inflammation during early pneumonia | 2016 |
| ^20^ | *Gzma×b-/-* | Gzma and B are critical for Tc/NK granule–mediated nucleolysis, with Gzma being the main contributor | 1997 |
| ^21^ | *Gzma×b-/-* | Virus-induced liver damage only occurs when both the FasL/Fas and the perforin pathways, including Gzma and B, are simultaneously activated | 2001 |
| ^22^ | *Gzma-/-*,  *Gzmb-/-*  *Gzma×b-/-* | Gzma and Gzma induce rapid perf‐mediated apoptosis | 2002 |
| ^23^ | *Gzma×b-/-* | Fas x GzmaxB-/-mice, in contrast to B6, Fas-/-, and GzmaxB-/- mice, do not recover from a primary infection with LCMV | 2004 |
| ^24^ | *Gzma-/-*, *Gzmb-/-* | Gzma and Gzma induce multiple independent cell death pathways | 2004 |
| ^25^ | *Gzma-/-* *Gzmb-/-* , Gzmaxb-/- | Gzma and Gzma are important in controlling replicating virus | 2006 |
| ^26^ | *Gzma×b-/-* | Gzma- and B-cluster deficiency delays the acute progression of pneumovirus disease by reducing alveolar injury. | 2010 |
| ^27^ | *Gzma-/-* *Gzmb-/-*, Gzmaxb-/- | Gzma/B deficiency associated with Th2 cytokine and Ab shift, enhanced early inflammatory gene expression. Gzma deficiency linked with reduced inflammation | 2011 |
| ^28^ | Gzmaxb-/- | Impaired control of parasite replication and reduced mononuclear cell recruitment in GzmaxB DKO mice in early-stage liver infection by Leishmania | 2015 |
| ^29^ | Gzmaxb-/- | Failed target cell death and delayed detachment of the killer cell causes significant increase in cytokine release in OTI Gzmab-/- CTLs | 2015 |
| **Studies showing no phenotype** | | | |
| ^30^ | *Gzma-/-*, *Gzmb-/-*, *Gzma×b-/-* | The course of Leishmania major infection in mice lacking granzyme-mediated mechanisms | 2002 |
| ^31^ | *Gzma-/-*, *Gzmb-/-*, *Gzma×b-/-* | Gzma and B are not essential for perforin-mediated tumor rejection | 2003 |
| ^32^ | *Gzma×b-/-* | Concerted action of perforin and granzymes is critical for the elimination of Trypanosoma cruzi from mouse tissues, but prevention of early host death is in addition dependent on the FasL/Fas pathway | 2003 |
| ^33^ | *Gzma-/-*, *Gzma×b-/-* | Fas-mediated inhibition of CD4+ T cell priming results in dominance of type 1 CD8+ T cells in the immune response to the contact sensitizer trinitrophenyl | 2004 |
| ^34^ | *Gzma-/-*, *Gzmb-/-*, *Gzma×b-/-* | NK cell-mediated immunopathology during an acute viral infection of the CNS | 2006 |
| ^35^ | *Gzma-/-*, *Gzma×b-/-* | Gzma-induced cell death exerted by ex vivo CTL: discriminating requirements for cell death and some of its signs. | 2008 |
| ^36^ | *Gzma×b-/-* | Rapid and efficient in vivo cytotoxicity by cytotoxic T cells is independent of Gzma and B | 2009 |
| ^37^ | *Gzma-/-*, *Gzmb-/-* | Characterizing the anti-tumor function of adoptively transferred NK cells in vivo | 2010 |
| ^38^ | *Gzma-/-*, *Gzmb-/-*, *Gzma×b-/-* | Cathepsin C limits acute viral infection independently of NK cell and CD8+ T-cell cytolytic function | 2011 |
| ^39^ | *Gzma-/-* | Gzma is dispensable in the development of diabetes in non-obese diabetic mice | 2012 |
| ^40^ | *Gzma-/-* | Gzma Is Expressed in Mouse Lungs during Mycobacterium tuberculosis Infection but Does Not Contribute to Protection In Vivo | 2016 |

**References**

1 Mullbacher, A. *et al.* Granzyme A is critical for recovery of mice from infection with the natural cytopathic viral pathogen, ectromelia. *Proc Natl Acad Sci U S A* **93**, 5783-5787, doi:10.1073/pnas.93.12.5783 (1996).

2 Mullbacher, A., Hla, R. T., Museteanu, C. & Simon, M. M. Perforin is essential for control of ectromelia virus but not related poxviruses in mice. *J Virol* **73**, 1665-1667 (1999).

3 Shresta, S., Graubert, T. A., Thomas, D. A., Raptis, S. Z. & Ley, T. J. Granzyme A initiates an alternative pathway for granule-mediated apoptosis. *Immunity* **10**, 595-605, doi:10.1016/s1074-7613(00)80059-x (1999).

4 Pereira, R. A., Simon, M. M. & Simmons, A. Granzyme A, a noncytolytic component of CD8(+) cell granules, restricts the spread of herpes simplex virus in the peripheral nervous systems of experimentally infected mice. *J Virol* **74**, 1029-1032, doi:10.1128/jvi.74.2.1029-1032.2000 (2000).

5 Loh, J., Thomas, D. A., Revell, P. A., Ley, T. J. & Virgin, H. W. t. Granzymes and caspase 3 play important roles in control of gammaherpesvirus latency. *J Virol* **78**, 12519-12528, doi:10.1128/JVI.78.22.12519-12528.2004 (2004).

6 Metkar, S. S. *et al.* Human and mouse granzyme A induce a proinflammatory cytokine response. *Immunity* **29**, 720-733, doi:10.1016/j.immuni.2008.08.014 (2008).

7 Velaga, S. *et al.* Granzyme A Is Required for Regulatory T-Cell Mediated Prevention of Gastrointestinal Graft-versus-Host Disease. *PLoS One* **10**, e0124927, doi:10.1371/journal.pone.0124927 (2015).

8 van den Boogaard, F. E. *et al.* Granzyme A impairs host defense during Streptococcus pneumoniae pneumonia. *American journal of physiology. Lung cellular and molecular physiology* **311**, L507-516, doi:10.1152/ajplung.00116.2016 (2016).

9 Garcia-Laorden, M. I. *et al.* Expression and Function of Granzymes A and B in Escherichia coli Peritonitis and Sepsis. *Mediators Inflamm* **2017**, 4137563, doi:10.1155/2017/4137563 (2017).

10 Mollah, Z. U. A. *et al.* Granzyme A Deficiency Breaks Immune Tolerance and Promotes Autoimmune Diabetes Through a Type I Interferon-Dependent Pathway. *Diabetes* **66**, 3041-3050, doi:10.2337/db17-0517 (2017).

11 Santiago, L. *et al.* Granzyme A Contributes to Inflammatory Arthritis in Mice Through Stimulation of Osteoclastogenesis. *Arthritis Rheumatol* **69**, 320-334, doi:10.1002/art.39857 (2017).

12 Park, S. *et al.* Granzyme A-producing T helper cells are critical for acute graft-versus-host disease. *JCI Insight* **5**, e124465, doi:10.1172/jci.insight.124465 (2020).

13 Santiago, L. *et al.* Extracellular Granzyme A Promotes Colorectal Cancer Development by Enhancing Gut Inflammation. *Cell Rep* **32**, 107847, doi:10.1016/j.celrep.2020.107847 (2020).

14 Susanto, O. *et al.* Mouse granzyme A induces a novel death with writhing morphology that is mechanistically distinct from granzyme B-induced apoptosis. *Cell Death Differ* **20**, 1183-1193, doi:10.1038/cdd.2013.59 (2013).

15 Arias, M. A. *et al.* Elucidating sources and roles of granzymes A and B during bacterial infection and sepsis. *Cell Rep* **8**, 420-429, doi:10.1016/j.celrep.2014.06.012 (2014).

16 Mullbacher, A. *et al.* Granzymes are the essential downstream effector molecules for the control of primary virus infections by cytolytic leukocytes. *Proc Natl Acad Sci U S A* **96**, 13950-13955, doi:10.1073/pnas.96.24.13950 (1999).

17 Riera, L. *et al.* Murine cytomegalovirus replication in salivary glands is controlled by both perforin and granzymes during acute infection. *Eur J Immunol* **30**, 1350-1355, doi:10.1002/(SICI)1521-4141(200005)30:5<1350::AID-IMMU1350>3.0.CO;2-J (2000).

18 Pardo, J., Balkow, S., Anel, A. & Simon, M. M. Granzymes are essential for natural killer cell-mediated and perf-facilitated tumor control. *Eur J Immunol* **32**, 2881-2887, doi:10.1002/1521-4141(2002010)32:10<2881::AID-IMMU2881>3.0.CO;2-K (2002).

19 Garcia-Laorden, M. I. *et al.* Granzymes A and B Regulate the Local Inflammatory Response during Klebsiella pneumoniae Pneumonia. *J Innate Immun* **8**, 258-268, doi:10.1159/000443401 (2016).

20 Simon, M. M. *et al.* In vitro- and ex vivo-derived cytolytic leukocytes from granzyme A x B double knockout mice are defective in granule-mediated apoptosis but not lysis of target cells. *J Exp Med* **186**, 1781-1786, doi:10.1084/jem.186.10.1781 (1997).

21 Balkow, S. *et al.* Concerted action of the FasL/Fas and perforin/granzyme A and B pathways is mandatory for the development of early viral hepatitis but not for recovery from viral infection. *J Virol* **75**, 8781-8791, doi:10.1128/jvi.75.18.8781-8791.2001 (2001).

22 Pardo, J., Balkow, S., Anel, A. & Simon, M. M. The differential contribution of granzyme A and granzyme B in cytotoxic T lymphocyte-mediated apoptosis is determined by the quality of target cells. *Eur J Immunol* **32**, 1980-1985, doi:10.1002/1521-4141(200207)32:7<1980::AID-IMMU1980>3.0.CO;2-Z (2002).

23 Rode, M. *et al.* Perforin and Fas act together in the induction of apoptosis, and both are critical in the clearance of lymphocytic choriomeningitis virus infection. *J Virol* **78**, 12395-12405, doi:10.1128/JVI.78.22.12395-12405.2004 (2004).

24 Pardo, J. *et al.* Apoptotic pathways are selectively activated by granzyme A and/or granzyme B in CTL-mediated target cell lysis. *J Cell Biol* **167**, 457-468, doi:10.1083/jcb.200406115 (2004).

25 van Dommelen, S. L. *et al.* Perforin and granzymes have distinct roles in defensive immunity and immunopathology. *Immunity* **25**, 835-848, doi:10.1016/j.immuni.2006.09.010 (2006).

26 Bem, R. A. *et al.* Granzyme A- and B-cluster deficiency delays acute lung injury in pneumovirus-infected mice. *J Immunol* **184**, 931-938, doi:10.4049/jimmunol.0903029 (2010).

27 Hartmann, W. *et al.* A novel and divergent role of granzyme A and B in resistance to helminth infection. *J Immunol* **186**, 2472-2481, doi:10.4049/jimmunol.0902157 (2011).

28 Murray, H. W., Mitchell-Flack, M., Zheng, H. & Ma, X. Granzyme-mediated regulation of host defense in the liver in experimental Leishmania donovani infection. *Infect Immun* **83**, 702-712, doi:10.1128/IAI.02418-14 (2015).

29 Jenkins, M. R. *et al.* Failed CTL/NK cell killing and cytokine hypersecretion are directly linked through prolonged synapse time. *J Exp Med* **212**, 307-317, doi:10.1084/jem.20140964 (2015).

30 Eisert, V., Munster, U., Simon, M. M. & Moll, H. The course of Leishmania major infection in mice lacking granzyme-mediated mechanisms. *Immunobiology* **205**, 314-320, doi:10.1078/0171-2985-00134 (2002).

31 Smyth, M. J., Street, S. E. & Trapani, J. A. Cutting edge: granzymes A and B are not essential for perforin-mediated tumor rejection. *J Immunol* **171**, 515-518, doi:10.4049/jimmunol.171.2.515 (2003).

32 Muller, U. *et al.* Concerted action of perforin and granzymes is critical for the elimination of Trypanosoma cruzi from mouse tissues, but prevention of early host death is in addition dependent on the FasL/Fas pathway. *Eur J Immunol* **33**, 70-78, doi:10.1002/immu.200390009 (2003).

33 Martin, S. F. *et al.* Fas-mediated inhibition of CD4+ T cell priming results in dominance of type 1 CD8+ T cells in the immune response to the contact sensitizer trinitrophenyl. *J Immunol* **173**, 3178-3185, doi:10.4049/jimmunol.173.5.3178 (2004).

34 Alsharifi, M. *et al.* NK cell-mediated immunopathology during an acute viral infection of the CNS. *Eur J Immunol* **36**, 887-896, doi:10.1002/eji.200535342 (2006).

35 Pardo, J. *et al.* Granzyme B-induced cell death exerted by ex vivo CTL: discriminating requirements for cell death and some of its signs. *Cell Death Differ* **15**, 567-579, doi:10.1038/sj.cdd.4402289 (2008).

36 Regner, M. *et al.* Cutting edge: rapid and efficient in vivo cytotoxicity by cytotoxic T cells is independent of granzymes A and B. *J Immunol* **183**, 37-40, doi:10.4049/jimmunol.0900466 (2009).

37 Pegram, H. J., Haynes, N. M., Smyth, M. J., Kershaw, M. H. & Darcy, P. K. Characterizing the anti-tumor function of adoptively transferred NK cells in vivo. *Cancer immunology, immunotherapy : CII* **59**, 1235-1246, doi:10.1007/s00262-010-0848-7 (2010).

38 Andoniou, C. E., Fleming, P., Sutton, V. R., Trapani, J. A. & Degli-Esposti, M. A. Cathepsin C limits acute viral infection independently of NK cell and CD8+ T-cell cytolytic function. *Immunol Cell Biol* **89**, 540-548, doi:10.1038/icb.2010.115 (2011).

39 Mollah, Z. U. *et al.* Granzyme B is dispensable in the development of diabetes in non-obese diabetic mice. *PLoS One* **7**, e40357, doi:10.1371/journal.pone.0040357 (2012).

40 Uranga, S., Marinova, D., Martin, C., Pardo, J. & Aguilo, N. Granzyme A Is Expressed in Mouse Lungs during Mycobacterium tuberculosis Infection but Does Not Contribute to Protection In Vivo. *PLoS One* **11**, e0153028, doi:10.1371/journal.pone.0153028 (2016).
