# Supplementary material for: Widespread discrepancy in Nnt genotypes and genetic backgrounds complicates granzyme A and other knockout mouse studies
Source: eLife. 2022 Feb 4;11:e70207. doi: 10.7554/eLife.70207 (PMC8816380; doi:10.7554/eLife.70207)
Supplement: Supplementary file 3. — Genetic differences potentially involved in inflammation or arthritis are indicated. Nnt not included. Differences in introns not included. [file elife-70207-supp3.docx]

**Supplementary File 3.** Genetic differences between *Gzma*^-/-^ and C57BL/6J; those potentially involved in inflammation or arthritis are indicated. NNT not included. Differences in introns not included.

| **Chromosome** | **Gene** | **Genetic difference*** | **Full gene name and canonical gene function** | **Reported role in inflammation/arthritis** |
| --- | --- | --- | --- | --- |
| 13 | Spata31 | NP | Spermatogenesis-associated protein 31. Testes specific gene involved in spermatogenesis ^1^. | *None found* |
| 1 | Bmpr2 | MP | Bone Morphogenetic Protein Receptor Type 2. Serine/threonine kinase receptor involved in embrogenesis and bone development ^2^. | Bmpr2 inhibits IL-34 and may antagonise inflammation and bone erosions in rheumatoid arthritis ^3^. |
| 4 | Srfs18/Pnisr | MP | PNN Interacting Serine and Arginine Rich Protein. | *None found* |
| 4 | Hp1bp3 | MP | Heterochromatin protein 1-binding protein 3. Transcriptional regulation during cell cycle ^4^. | - Hp1bp3 knockdown activated neuroinflammatory-related pathways ^5^. |
| 4 | Padi3 | MP | Peptidyl arginine deiminase 3. Deimination of arginine residues of proteins in epidermis and hair follicles ^6^. | *None found* |
| 4 | Casz1 | MP | Zinc finger protein castor homolog 1. Transcription factor important in neuronal, retinal, vasculature and cardiomyocyte differentiation and development ^7^. | - Casz1 is expressed in CD4^+^ T cells and is involved in Th17 differentiation and important in determining Th1/Th17 balance ^7^. |
| 5 | Adamts3 | MP | - ADAM Metallopeptidase With Thrombospondin Type 1 Motif 3. Involved in collagen fibril formation ^8^. | - ADAMTS-3 expression is reported to be increased in OA cartilage ^8^. |
| 5 | Fras1 | MP | Fraser extracellular matrix complex subunit 1. Involved in cell adhesion and intercellular signalling during embryonic development ^9^. | *None found* |
| 5 | Myo18b | MP | Myosin XVIIIb. Myofibril organization during skeletal and cardiac muscle development ^10^. | Down-regulated in a collagen-induced arthritis, involved in skeletal muscle proliferation, cell cycle and migration and differentiation of myoblasts ^11^. |
| 6 | Mkrn1 | MP | Makorin ring finger protein 1. An E3 ligase of PPARγ that induces its ubiquitination, followed by proteasomal degradation ^12^. | Stable overexpression of MKRN1 reduced PPAR*γ* protein levels and suppressed adipocyte differentiation in 3T3-L1 and C3H10T1/2 cells ^12^. |
| 7 | Nlrp12 | MP | NLR family pyrin domain containing 12, encodes a member of the CATERPILLER family of cytoplasmic proteins. Functions as an attenuating factor of inflammation by suppressing inflammatory responses in activated monocytes. Mutations in this gene cause familial cold autoinflammatory syndrome type 2. | - This mutation has been shown to cause a neutrophil recruitment defect in 6J mice ^13^. Reduces RIG-I induction of interferon and cytokine responses to RNA viruses by interaction with TRIM25 ^14^.   Regulates NF-KB, inflammasome, dendritic cell migration & transcription of MHC class I genes ^15^. |
| 7 | Herc2 | MP | - HECT and RLD domain containing E3 ubiquitin protein ligase 2. | A marker of inflammation  ^16^. HERC2 loci implicated in inflammatory bowel disease and type 1 diabetes ^17^ and Crohn's disease ^18^. |
| 7 | Acan | MP | A member of the aggrecan/versican proteoglycan family. The encoded protein is an integral part of the extracellular matrix in cartilagenous tissue. | Involved in rheumatoid arthritis ^19^. Mutations in this gene may be involved in skeletal dysplasia and spinal degeneration. |
| 7 | Olfr577 | MP | Olfactory receptor | *None found* |
| 10 | Jmjd1c | MP | The protein interacts with thyroid hormone receptors and contains a jumonji domain. It is a candidate histone demethylase and is thought to be a coactivator for key transcription factors. | - JMJD1C loss leads to increased IL-3 signaling. This effect is blunted in leukemia cells with activating RAS mutations ^20^. |
| 11 | Cyfip2 | MP | Involved in T-cell adhesion and p53/TP53-dependent induction of apoptosis. | Upregulated in rheumatoid arthritis patients ^21^ |
| 13 | Ndufs6 | MP | Encodes a subunit of the NADH:ubiquinone oxidoreductase (complex I). | Complex I deficiency can result in increased inflammation due to the generation of reactive oxygen species by innate immune cells ^22^ |
| 13 | NkapI | MP | *None found* | *None found* |
| 14 | Fam160b2/ RAI16 | MP | Family with sequence similarity 160, member B2.  RAI16 is a novel A-kinase anchoring protein (AKAP). Regulates HSP70 associated anti-apoptosis signaling ^23^. | RAI16 may function to affect genes involved in intestinal barrier function and thus immunoprotective against inflammation ^24^. |
| 15 | Apol11b | MP | Apolipoprotein L 11b | *None found* |
| 17 | Guca1a | MP | Guanylyl cyclase-activating protein 1. Encodes an enzyme that plays a role in the recovery of retinal photoreceptors from photobleaching. | *None found* |
| X | Armcx4 | MP | - Belongs to the armadillo repeat-containing family of proteins, which interact with other proteins in a variety of cellular processes. | *None found* |
| 5 | Tbc1d19 | SSP | May act as a GTPase-activating protein for Rab family protein(s). | *None found* |
| 1 | Crb1 | Frameshift | - An essential role in normal vision. This protein is found in the brain and the retina. | *None found* |
| 9 | Cilp | Frameshift | Cartilage intermediate layer protein. | Increases with ageing and early osteoarthritis ^25^. Autoantigen in osteoarthritis & rheumatoid arthritis   - Immunization with CILP induces a mild but chronic arthropathy in mice ^26^. Clip inhibits TGF-β1 ^27^. |

*** Genetic difference;** Nonsense polymorphism (NP) is introduction of a premature stop codon. Missense polymorphism (MP) is a single amino acid change. Splice site polymorphism (SSP). Frameshift = 1bp deletion.

**References**

1. Wu, Yang, Xu, Yu, *Mol Reprod Dev* **82**, 432-440 (2015).

2. Gilboa *et al.*, *Molecular biology of the cell* **11**, 1023-1035 (2000).

3. Chemel *et al.*, *Am J Pathol* **187**, 156-162 (2017).

4. Dutta *et al.*, *Molecular &amp;amp; Cellular Proteomics* **13**, 2183 (2014).

5. Neuner, Ding, Kaczorowski, *Aging Cell* **18**, e12886 (2019).

6. Nishijyo *et al.*, *J Biochem* **121**, 868-875 (1997).

7. Bhaskaran *et al.*, *Front Immunol* **9**, 184 (2018).

8. Yang, Chanalaris, Troeberg, *Osteoarthritis and cartilage* **25**, 1000-1009 (2017).

9. Jordan *et al.*, *Human Molecular Genetics* **27**, 2064-2075 (2018).

10. Latham *et al.*, *Cell Reports* **32**, 108090 (2020).

11. Tang *et al.*, *Mol Med Rep* **20**, 4843-4854 (2019).

12. Kim *et al.*, *Cell Death Differ* **21**, 594-603 (2014).

13. Ulland *et al.*, *Nat Commun* **7**, 13180 (2016).

14. Chen *et al.*, *Cell Host Microbe* **25**, 602-616.e607 (2019).

15. Tuncer, Fiorillo, Sorrentino, *J Leukoc Biol* **96**, 991-1000 (2014).

16. Garcia-Heredia, Carnero, *Oncotarget* **8**, 98580-98597 (2017).

17. Wang *et al.*, *Hum Mol Genet* **19**, 2059-2067 (2010).

18. Weersma *et al.*, *Am J Gastroenterol* **104**, 630-638 (2009).

19. Hanyecz *et al.*, *Biomed Res Int* **2014**, 942148 (2014).

20. Izaguirre-Carbonell *et al.*, *Blood Adv* **3**, 1499-1511 (2019).

21. Afroz *et al.*, *Front Immunol* **8**, 74 (2017).

22. Ng *et al.*, *Helicobacter* **22**, (2017).

23. Ding *et al.*, *Oncotarget* **6**, 15540-15550 (2015).

24. Xu *et al.*, *Cell Death Dis* **10**, 958 (2019).

25. Tsuruha *et al.*, *Arthritis Rheum* **44**, 838-845 (2001).

26. Yao *et al.*, *Annals of the Rheumatic Diseases* **63**, 252 (2004).

27. van Nieuwenhoven *et al.*, *Scientific Reports* **7**, 16042 (2017).
