# Supplementary figures and images for: Widespread discrepancy in Nnt genotypes and genetic backgrounds complicates granzyme A and other knockout mouse studies
Source: eLife. 2022 Feb 4;11:e70207. doi: 10.7554/eLife.70207 (PMC8816380; doi:10.7554/eLife.70207)

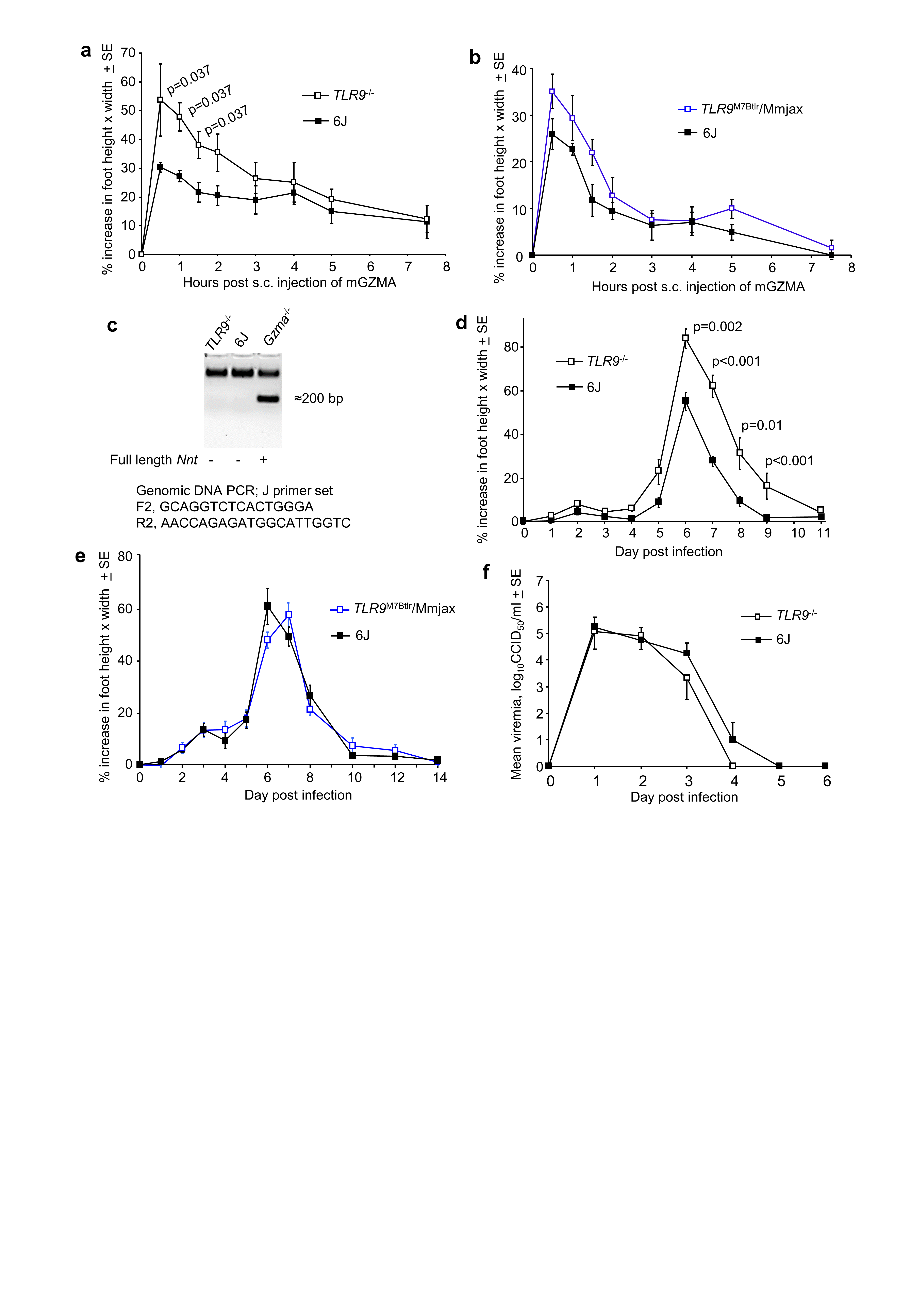

Supplement: Supplementary file 8. — (a) Tlr9-/- and 6J mice were injected intraplantar into the feet with 5 µg recombinant mouse granzyme A (GZMA) in 20 µl and foot swelling measured over time as described (Schanoski et al., 2019) (n = 4 mice and four feet per group; statistics by Kolmogorov–Smirnov tests). Tlr9-/- mice were derived from 129/Ola × C57BL/6F1 progeny (http://www.myv.ne.jp/obs/index.files/tlr_eng.htm). (b) As for (a) using Tlr9M7Btlr/Mmjax and 6J mice. Tlr9M7Btlr/Mmjax mice have a Tlr9 missense point mutation and do not respond to oligonucleotides containing CpG motifs (https://www.jax.org/strain/014534). (c) Tlr9-/- mice (like 6J) do not encode the full Nnt gene. (d) Female 8–10-week-old Tlr9-/- and 6J mice (n = 6 mice and 12 feet per group) were infected with chikungunya virus (CHIKV) and feet measured over time. Statistics by Kolmogorov–Smirnov tests. (e) Female C57BL/6J-Tlr9M7Btlr/Mmjax mice (n = 6 mice and 12 feet per group) were infected as for (d). (f) Viremia for the mice in (d). After GZMA injection, Tlr9-/- mice showed increased foot swelling (a), whereas C57BL/6J-Tlr9M7Btlr/Mmjax mice showed no significant difference (b). Tlr9-/- mice also have the Nnt deletion (c); however, they are on a mixed 129/Ola and C57BL/6 background (Hemmi et al. 2000), with 129/SvJ mice showing increased inflammatory infiltrates in certain settings (Hoover-Plow et al., 2008). After CHIKV infection, foot swelling was again increased in Tlr9-/- (d), but not Tlr9M7Btlr/Mmjax mice (e). Tlr9-/- mice did not show an increased viremia (f). These data do not support a contention that TLR9 is required for GZMA’s bioactivity. [file elife-70207-supp8.zip › Supplementary File 8.TIF]

## Slide 1
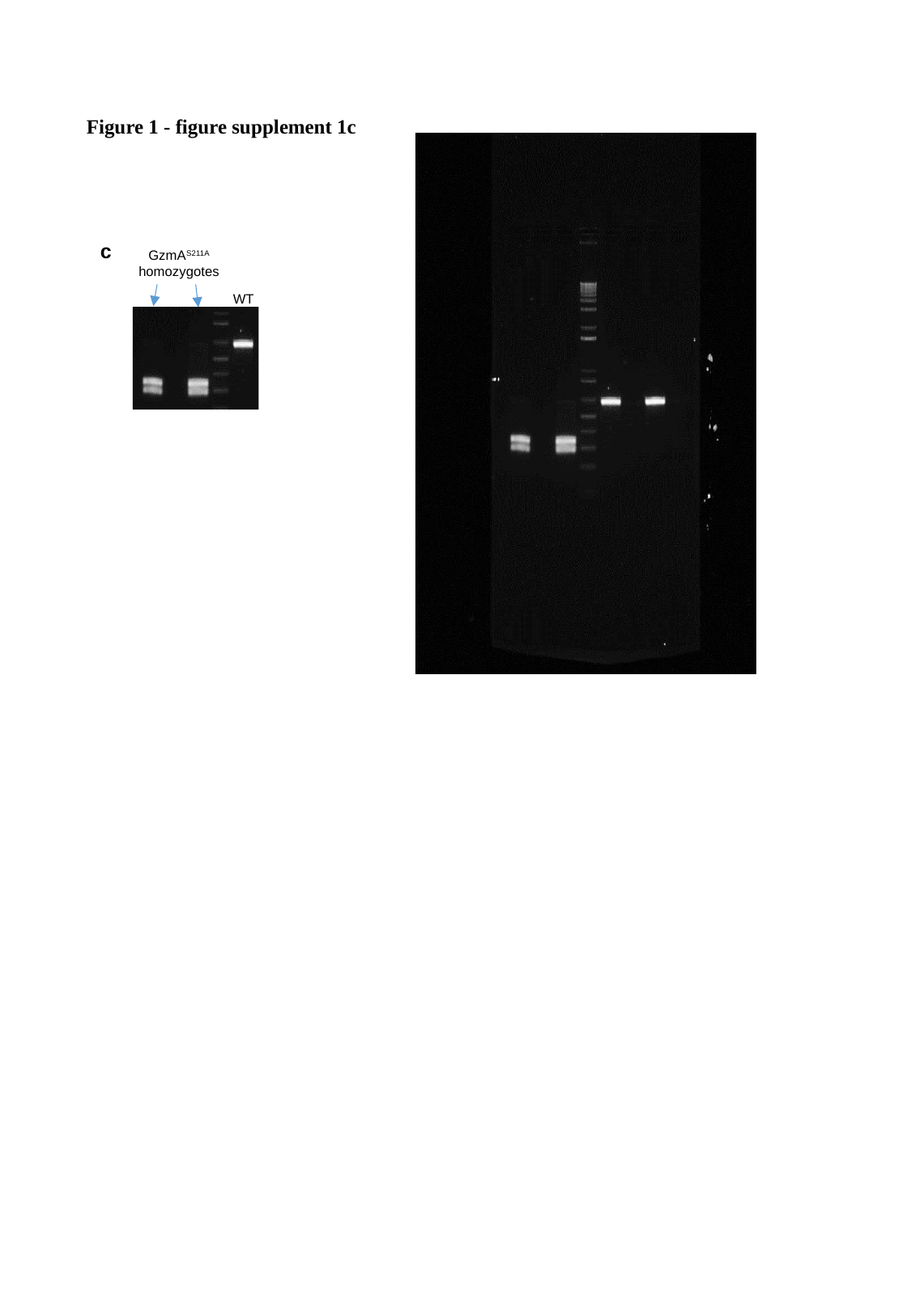

Figure 1 - figure supplement 1c
c
GzmAS211A
homozygotes
WT

Supplement: Source data 1. — Source data for DNA gel images in Figure 3e, Figure 1—figure supplement 1b,c, and Figure 4—figure supplement 1e. [file elife-70207-supp9.zip › Source_data_File_1/Figure 1 - figure supplement 1c-source data 1.pptx]

## Slide 1
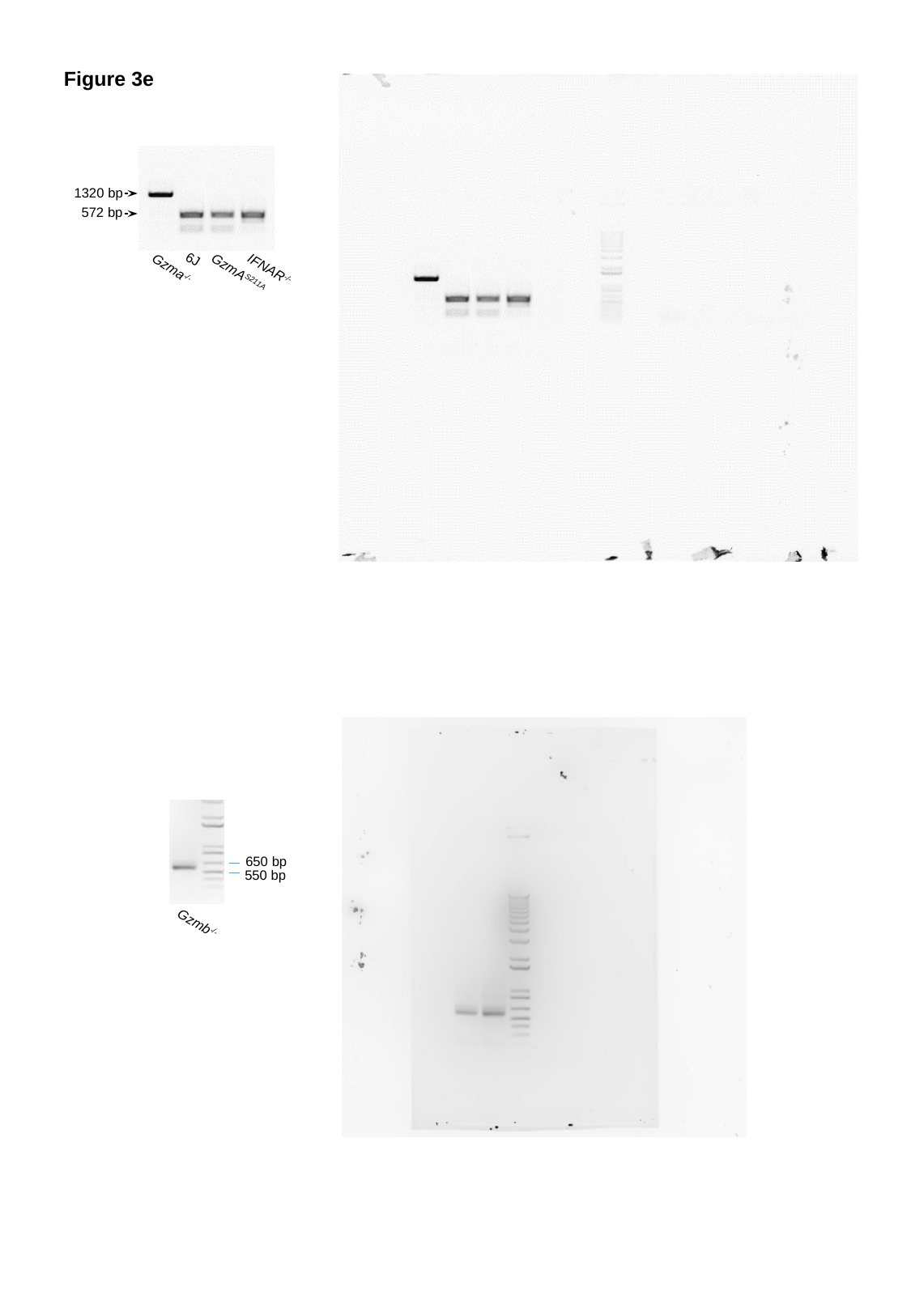

Figure 3e
1320 bp
572 bp
6J
Gzma-/-
IFNAR-/-
GzmAS211A
650 bp
550 bp
Gzmb-/-

Supplement: Source data 1. — Source data for DNA gel images in Figure 3e, Figure 1—figure supplement 1b,c, and Figure 4—figure supplement 1e. [file elife-70207-supp9.zip › Source_data_File_1/Figure 3e - source data 1.pptx]
