# Supplementary material for: Widespread discrepancy in Nnt genotypes and genetic backgrounds complicates granzyme A and other knockout mouse studies
Source: eLife. 2022 Feb 4;11:e70207. doi: 10.7554/eLife.70207 (PMC8816380; doi:10.7554/eLife.70207)
Supplement: Source data 1. — Source data for DNA gel images in Figure 3e, Figure 1—figure supplement 1b,c, and Figure 4—figure supplement 1e. [file elife-70207-supp9.zip › Source_data_File_1/Australian Phenomics Network report-source data for Figure 1 - figure supplement 1.pdf]

GzmA Cas9/CRISPR genome editing

Genotyping strategy

The forward (5’ TCAGCTGCTTTTGCCTGTTTCA 3’) and reverse (5’ GAGAAAGTCCCCTGTCCTCGG 3’) genotyping primers will amplify a product of 628bp from the wild type modified alleles. BsaHI digestion of the PCR product amplified from the correctly modified allele will generate fragments of 290bp and 338bp. Sequence analysis was used to determine the exact nature of modification events.

The assay was performed using AccuStart II GelTrack PCR SuperMix (Quantabio)

Wild type allele

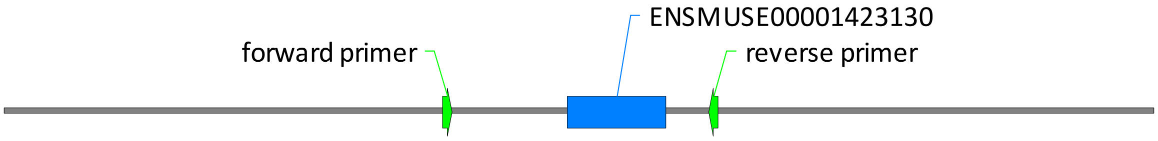

Reagents

2X reaction buffer	15.0μl
Forward primer (10μM)	1.2μl
Reverse primer (10μM)	1.2μl
Template	2.0μl
H_2O	9.4μl
**Total**	**30.0μl**

Cycling parameters

Denature	94°C	2min	x1 cycle
Denature	94°C	20sec	x35 cycles
Annealing	62°C	20sec	
Extension	72°C	45sec	
Extension	72°C	5min	x1 cycle

PCR result – progeny of ET44

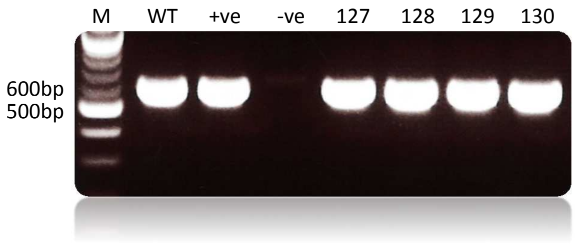

Restriction digest - BsaHI

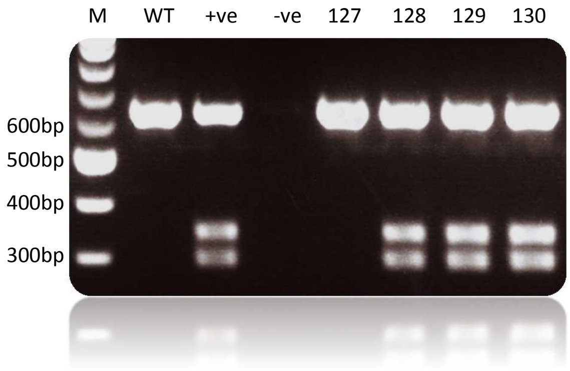

M	100bp ladder (NEB)
WT	wild type
+ve	positive control
-ve	no DNA control
127 – 130	A generation pups 127 – 130
