# Supplementary material for: Widespread discrepancy in Nnt genotypes and genetic backgrounds complicates granzyme A and other knockout mouse studies
Source: eLife. 2022 Feb 4;11:e70207. doi: 10.7554/eLife.70207 (PMC8816380; doi:10.7554/eLife.70207)

Nnt (C57BL/6J)

Gene: Nnt ENSMUSG00000025453

**Description**	nicotinamide nucleotide transhydrogenase [Source:MGI Symbol;Acc:MGI:109279]
**Gene Synonyms**	4930423F13Rik
**Location**	Chromosome 13: 119,335,448-119,408,997 reverse strand.<br>GRCm38:CM001006.2
**About this gene**	This gene has 5 transcripts (splice variants), 270 orthologues, 1 paralogue, is a member of 1 Ensembl protein family and is associated with 6 phenotypes.
**Transcripts**	Hide transcript table

Show/hide columns (1 hidden) Filter

Name	Transcript ID	bp	Protein	Biotype	CCDS	UniProt	Flags
Nnt-202	ENSMUST00000099149.9	3464	835aa	Protein coding	CCDS84067	Q8BGK0	TSL:1 GENCODE basic APPRIS P2
Nnt-203	ENSMUST00000109204.7	3198	713aa	Protein coding	-	E9Q8F4	TSL:5 GENCODE basic
Nnt-201	ENSMUST00000069902.12	3122	721aa	Protein coding	-	Q8C9V5	TSL:1 GENCODE basic APPRIS ALT2
Nnt-204	ENSMUST00000133627.7	2340	No protein	Retained intron	-	-	TSL:1
Nnt-205	ENSMUST00000144599.1	1885	No protein	Retained intron	-	-	TSL:1

Summary

**Name**	Nnt (MGI Symbol)
**CCDS**	This gene is a member of the Mouse CCDS set: CCDS84067.1
**Ensembl version**	ENSMUSG00000025453.18
**Gene type**	Protein coding
**Annotation method**	Annotation for this gene includes both automatic annotation from Ensembl and Havana manual curation, see article.
**Alternative genes**	**This gene corresponds to the following database identifiers:**<br>**Havana gene:** OTTMUSG00000021068

Nnt transcripts

C57BL/6J

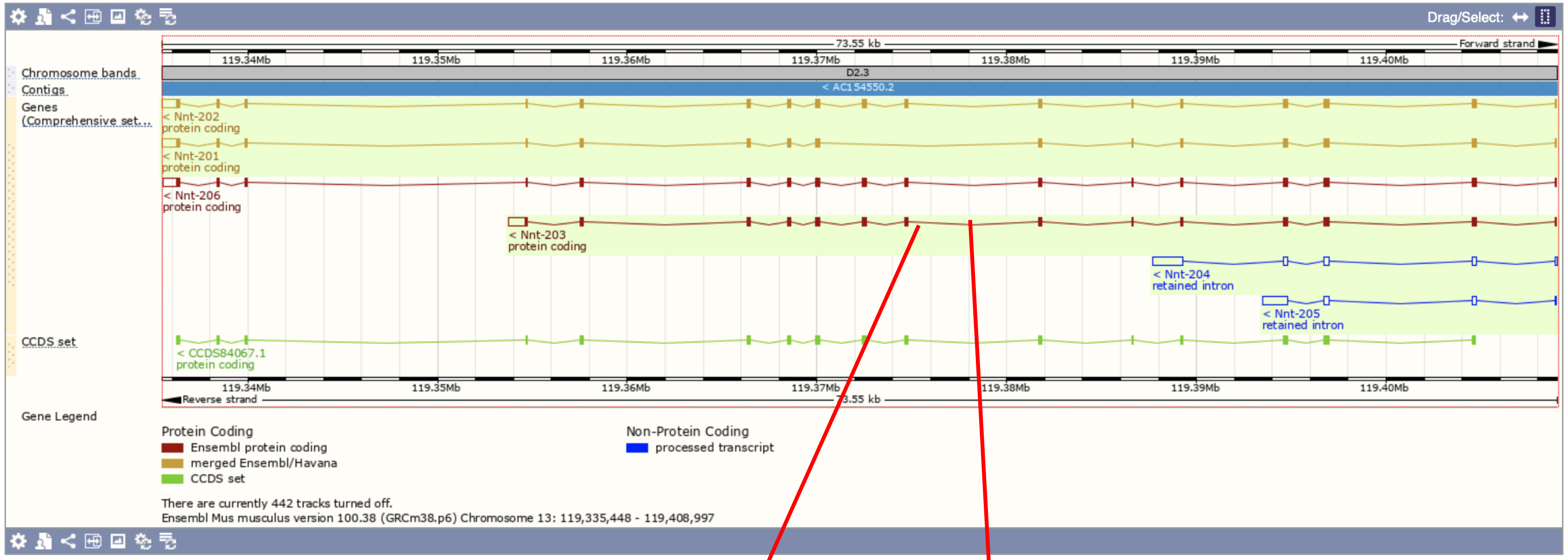

129
C57BL/6N

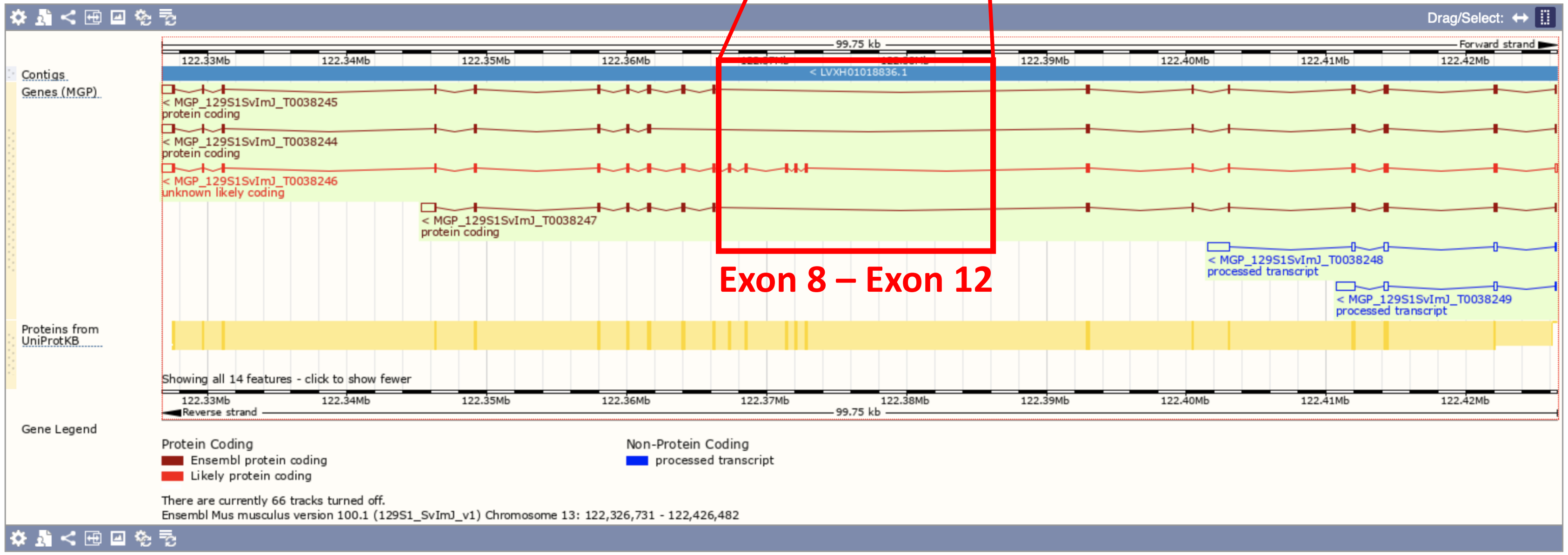

Nnt gene structures

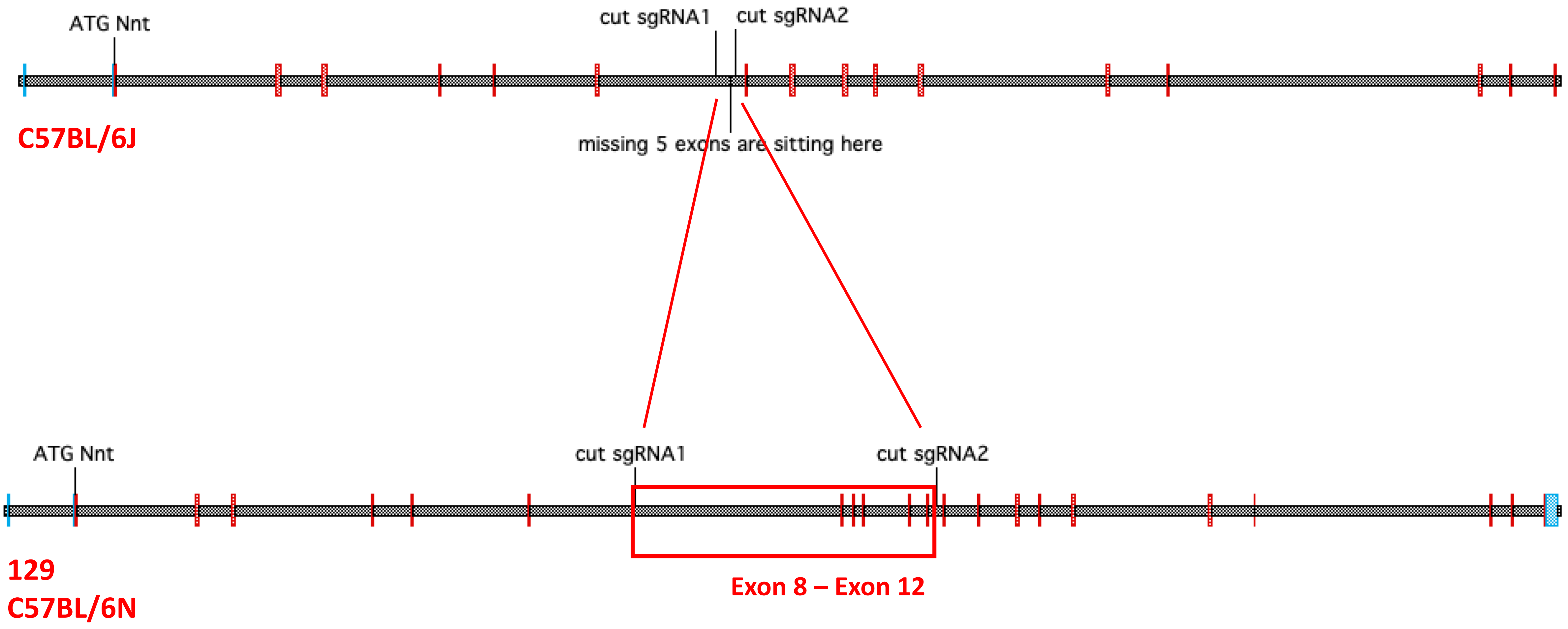

Positions of sgRNAs Nnt (shown on 6J genomic DNA)

```
                                                  cut sgRNA1
1    ATAGTCACTT TTATTCTTTA AAGGCTATCT AAAGAAAGCT GGACAGAACA CTCTGTTGGC TTTTTTTTTT
71   TTTTTTTTAA ATAGGATCAT TGTAAAAGCT AACTTAAGAC TGGAAACTTA GCTCTCTTAA ATTATCCAAA
141  AACTAAAACA CATGCCCCGT CTGCAATTTG AGGTGGCTAG CTTGACCTTT CAGGCACTGG AATTAGGAAG
211  TAGTGGAGGA TATTCAGGAA TTGTAAAGTG TCTTGTTGGG AAGGAAGATA TTCCTCGATT GTAAATTATA
281  ACATCTTAAT CCCGGTTAGA GAGCAGTATG TTTTGTGTTT ATCCTAGTTA GGGTCTATAA AATATCCCTG
351  TCACCGGACA CTTAAATCCT CCACAACTGG GGAAGAGTTG GGAAGGAGGT GTTTTCCTCT GATGGGACTC
421  AGCCTAAGAT GTTGCAGTCA CATAGAGCCA TAAATGATTC TGCCTCTGAT GCTGCCCATT TTTTCTTGAA
491  TGTATCTTCA TAGGATAGGA TGACAGTCTT GAAAAGATGG CATTGCAGAC AGGAGACTAG TCCAGCGGTA
561  ACTGGCACTT GCTGGTGGAA TTCCGCTGAG AGAACTCTTG CTCTGGCAAC TGTACAAAAG CCAGAAGAGT
631  TTATCATTCA GAGGGTTAGT TTCTTCTCTG TGCACATTAT GATTTTAGAA GTCAAAACTA CATTGTGTAG
701  GTCAGCAACT CCCAATGTTT TGGAAGTTGT ACTTCTTTTT TATTTTGAGT GTCTGATACG TCCTTCATGG
771  TCCCCTCCCT TCCATTTAGT TTATGACGCT ATCGAATTCT AAAATATTGC TTCCACACGA CCACTTATTC
                                                            missing 5 exons are sitting here
841  TGAGAATTCT GAAAGACAGC AAGAAATGCT TCGATGTTAC AACACTGCTT TGAGTGCAGA ACCAATCCTT
911  CCATACCCCC AGCCACACAG TAATGTTCTG CTAGAAGCTA ACTGGGAAGT CATGGAATGC ATAGTTTTGG
981  ATAACATTGA CTTCCACTGT CACCATGACA TTTGTAAATG AGTACAACTG CAGATAAGAT TTTAGTTTAT
1051 TGCCTTATCA CAATGTTGTA TGTTCCTGAA AACTGAAAGG CTAGCACATC ACTCATGTAT GACACTCATC
                                      cut sgRNA2
1121 TGATGCTATT TTATAGCTTA AAATTCCTTA GTGGGTCCAA GGTAACTGTT GATACAGTAT GTGTTCTTTA
1191 TCATTTGCAA CAGAGTTTGA TAAACAGAGT AACCTATCAG AGAAAAAATG CTGATTTTTG ACCC
```

Guides to delete exon 8 – exon 12 of Nnt (in 6N strain)

Nnt sgRNA1 CATGCCCCGT CTGCAATTTG
This guide cuts 6761 bp downstream of Exon 7 and 13210 bp upstream of Exon 8 of Nnt on C57BL/6N

Nnt sgRNA2 CAGTTACCTT GGACCCACTA
This guide cuts 527 bp downstream of Exon 12 of Nnt and 420 bp upstream of Exon 12 of Nnt on C57BL/6N

Nnt KO genotyping strategy

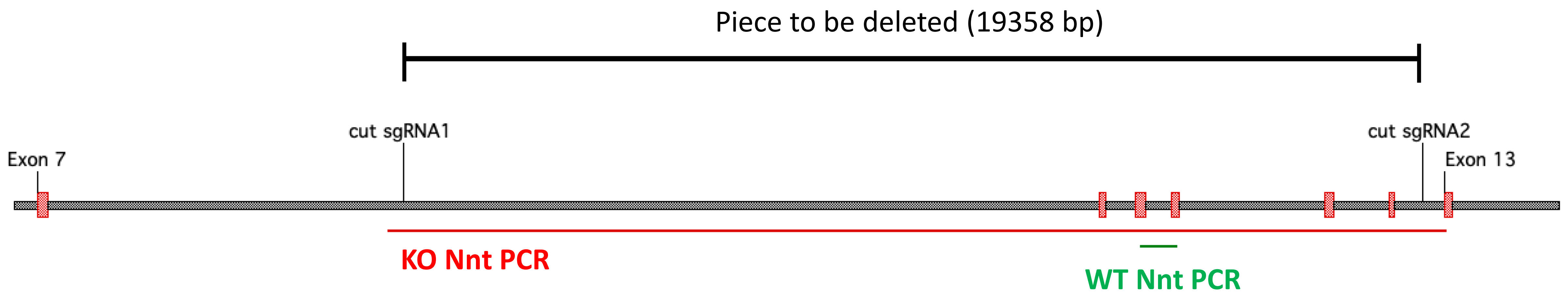

WT MICE:	WT PCR works / KO PCR doesn’t work
HETEROZYGOUS MICE:	WT PCR works / KO PCR works
HOMOZYGOUS MICE:	WT PCR doesn’t work / KO PCR works

WT genotyping PCR Nnt

PCR = 702 bp

part of exon 9

```
1   AG GCC ATC AGC CCT GAC AAG GAC AAT TTT CAC TTT GAA GTG AAG GAT GAC TTT GAC TTT GGT
P1 ------------------------>
63  ACA ATG AGT CAC GTC ATT CGA GGG ACC GTG GTG ATG AAG GTAGAGGTCC CCTTTTTTGT GTTCTTCCTG
132 TGACCCTGGA CTTAGGACTT CTGTCCTTGA AGAATTCCAT TATGGAATAG AGATGTGTTT GAATAAAGTA
202 TTGGCATGTC AGTGGAAATT GTGTTAAAAT GGAGAGTGAC TTAAGGAAAG CATTTCAAAG ACTCAAGTAT
272 GTATGAGTCA TTGAAAACAC ACCTCACTTT TGAAAACCAG GGGCAGAAAT TTAATAAAGT AAAAATGAAT
342 TGTTCTCCCC ATGTACATAC TTTTCAGGGT TTGAACAGTA TTCTTCTATA TGTGTCCCAG ACATACAAAA
412 ATTACATGTG TGCAGCACAA ACATTATTCA CTTATATACC CATTTTGTAA ATTATATGAA TTTATATGCA
482 TTAAAATTAT AGTTACAGTT TTTTTTCCAG CATGCACTCT CTTCTGTCAG TCATTTTGAA ATGCTAATTT
                                                Exon 10
552 GACTTTAAAA AACATTATTT CCTAATATTG GTTAG GAT GGC AAA GTG ATT TTC CCA GCT CCC ACA CCA
620 AAA AAT ATT CCC GAA GAA GCC CCA GTC AAA CCG AAG ACC GTG GCT GAG CTG GAA GCC GAG AAA
683 GCA GGC ACC GTC TCC ATG TA
    <------------------------ P2
```

→ = genotyping oligo

Red sequence shown is part of exon 9 and Exon 10 of Nnt, sequence in back is intronic sequence

Genotyping strategy - wild type PCR

Primers P1 (5’ AGGCCATCAGCCCTGACAAG 3’) and P2 (5’ TACATGGAGACGGTGCCTGC 3’) will amplify a product of 702bp from the wild type allele. Sequence analysis was used to determine the exact nature of modification events.

The assay was performed using AccuStart II GelTrack PCR SuperMix (Quantabio)

Reagents	
2X reaction buffer	15.0μl
Forward primer (10μM)	1.2μl
Reverse primer (10μM)	1.2μl
Template	2.0μl
Water	10.6μl
**Total**	**30.0μl**

Cycling parameters			
Denature	94°C	2min	x1 cycle
Denature	94°C	20sec	x35 cycles
Annealing	64°C	20sec	
Extension	72°C	60sec	
Extension	72°C	5min	x1 cycle

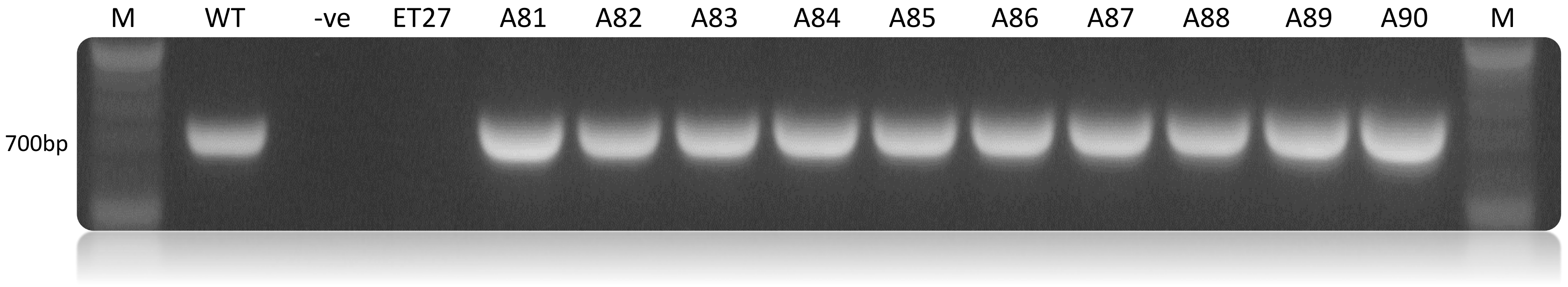

M	100bp ladder (NEB)
WT	wild type DNA
-ve	no DNA control
ET27	positive control (founder ET27)
A81 – A90	A generation pups

KO genotyping for Nnt

PCR = around 764 bp

(WT PCR band with these oligos is 20122 bp, this PCR won't work)

```
  1 ACAGGCTAAT CTGGGTGAGG CACTACCTCA TCTGAGATTC CCTCGTACTT CAAACACAGT AGTGGCTCAG
P3 ------------------------>
 71 CAAATATTGT TCAACTCATA ATCCTGTATG TGTTCCTTAG GTCTTGGTTG GCATAGTCAC TTTTATTCTT
141 TAAAGGCTAT CTAAAGAAAG CTGGACAGAA CACTCTGTTG GCTTTTTTTT TTTTTTTTTT TTAAATAGGA
211 TCATTGTAGA AGCTAACTTA AGACTGGAAA CTTAGCTCTC TTAAATTATC CAAAAACTAA AACACATGCC
               fusion between sgRNA1 and sgRNA2 Nnt
281 CCGTCTGCAA TTGGGTCCAA GGTAACTGTT GATACAGTAT GTGTTCTTTA TCATTTGCAA CAGAGTTTGA
351 TAAACAGAGT AACCTATCAG AGAAAAAATG CTGATTTTTG ACCCTTTGTA TTTTCAAGCC TTTAAATGGC
421 TATTTACTTT TAGTCATGCA GAAACAGTTG GCCCTACGTT CTGATAGCAA GTTTTCTTCC TAACTGTATC
491 TAGGGTTCCC CGTATACTTC ATCCACCTTA AGGGAACTGG CTTTAATAAT AGAGTGGCTT TTGCTTTTGA
561 GGTTCAAACT GTTACTTGAA ATAATAGCAT CACTTTGTAA TACTTAGTGA TAAAAGTATA CTTAAAATAA
631 ATATGCTGTA GCAGACGGAA AAAAGTCATG CTGTCTGACG TGACACCTCT TGCCTGTTTG TGCTTCTCTT
               Exon 13
701 GCTTCGGTAA GGT GGT TTT CTG GTG ACT CAG AGA ATG CTG GAC ATG TTC AAG CGA CCC ACG GAC
                                                        <------------------------- P4
```

→ = genotyping oligo

Genotyping strategy - knockout PCR

Primers P3 (5’ ACAGGCTAATCTGGGTGAGGC 3’) and P4 (5’ GTCCGTGGGTCGCTTGAACA 3’) will amplify a product of 764bp from the correctly modified allele. Sequence analysis was used to determine the exact nature of modification events.

The assay was performed using AccuStart II GelTrack PCR SuperMix (Quantabio)

**Reagents**	
2X reaction buffer	15.0μl
Forward primer (10μM)	1.2μl
Reverse primer (10μM)	1.2μl
Template	2.0μl
Water	10.6μl
**Total**	**30.0μl**

**Cycling parameters**			
Denature	94°C	2min	x1 cycle
Denature	94°C	20sec	x35 cycles
Annealing	64°C	20sec	
Extension	72°C	60sec	
Extension	72°C	5min	x1 cycle

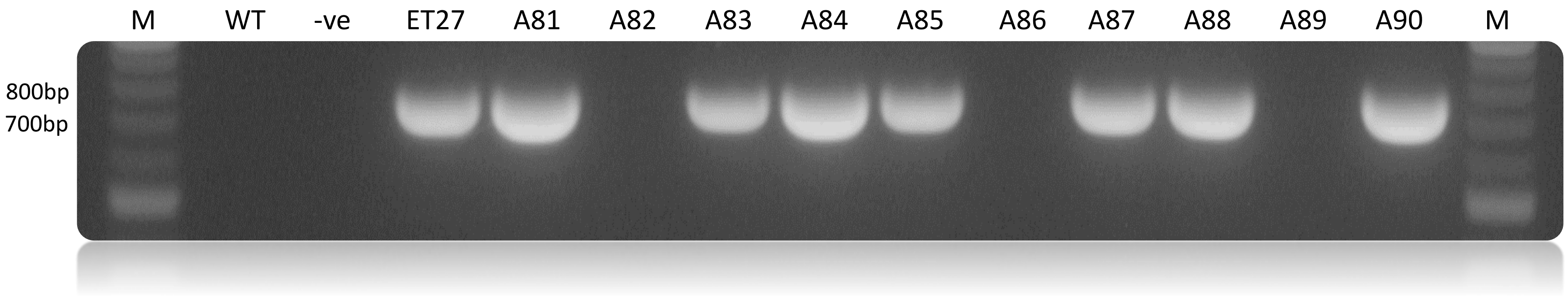

M	100bp ladder (NEB)
WT	wild type DNA
-ve	no DNA control
ET27	positive control (founder ET27)
A81 – A90	A generation pups

Genotyping strategy

Primer P4 (5’ GTCCGTGGGTCGCTTGAACA 3’) is used to sequence the knock out PCR product amplified using primers P3 and P4

Sequencing chromatogram for animal ET27

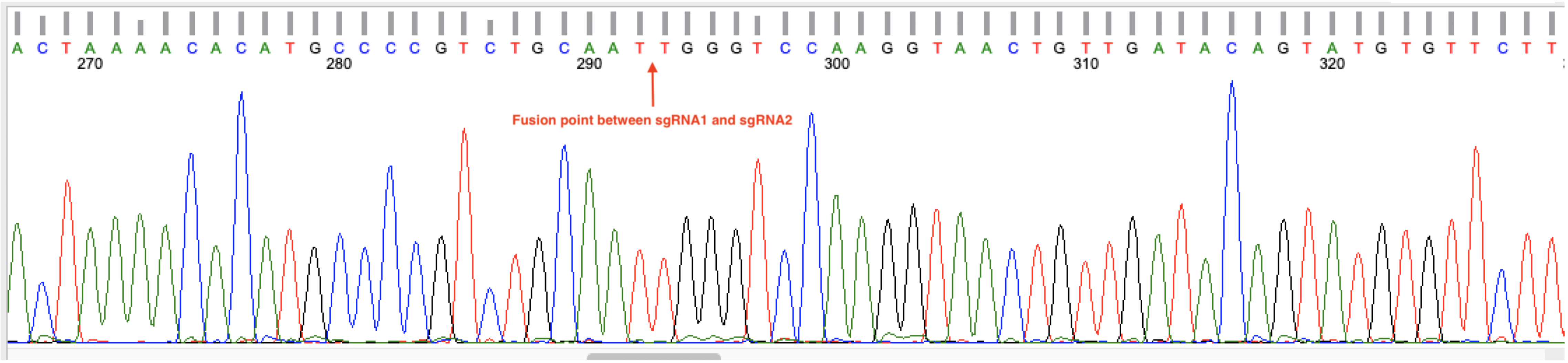

Supplement: Source data 1. — Source data for DNA gel images in Figure 3e, Figure 1—figure supplement 1b,c, and Figure 4—figure supplement 1e. [file elife-70207-supp9.zip › Source_data_File_1/Australian Phenomics Network report-source data for Figure 4 - figure supplement 1.pdf]
