# Supplementary material for: Widespread discrepancy in Nnt genotypes and genetic backgrounds complicates granzyme A and other knockout mouse studies
Source: eLife. 2022 Feb 4;11:e70207. doi: 10.7554/eLife.70207 (PMC8816380; doi:10.7554/eLife.70207)
Supplement: Source data 1. — Source data for DNA gel images in Figure 3e, Figure 1—figure supplement 1b,c, and Figure 4—figure supplement 1e. [file elife-70207-supp9.zip › Source_data_File_1/Figure 4 - figure supplement 1-source data 1.pptx]

## Slide 1
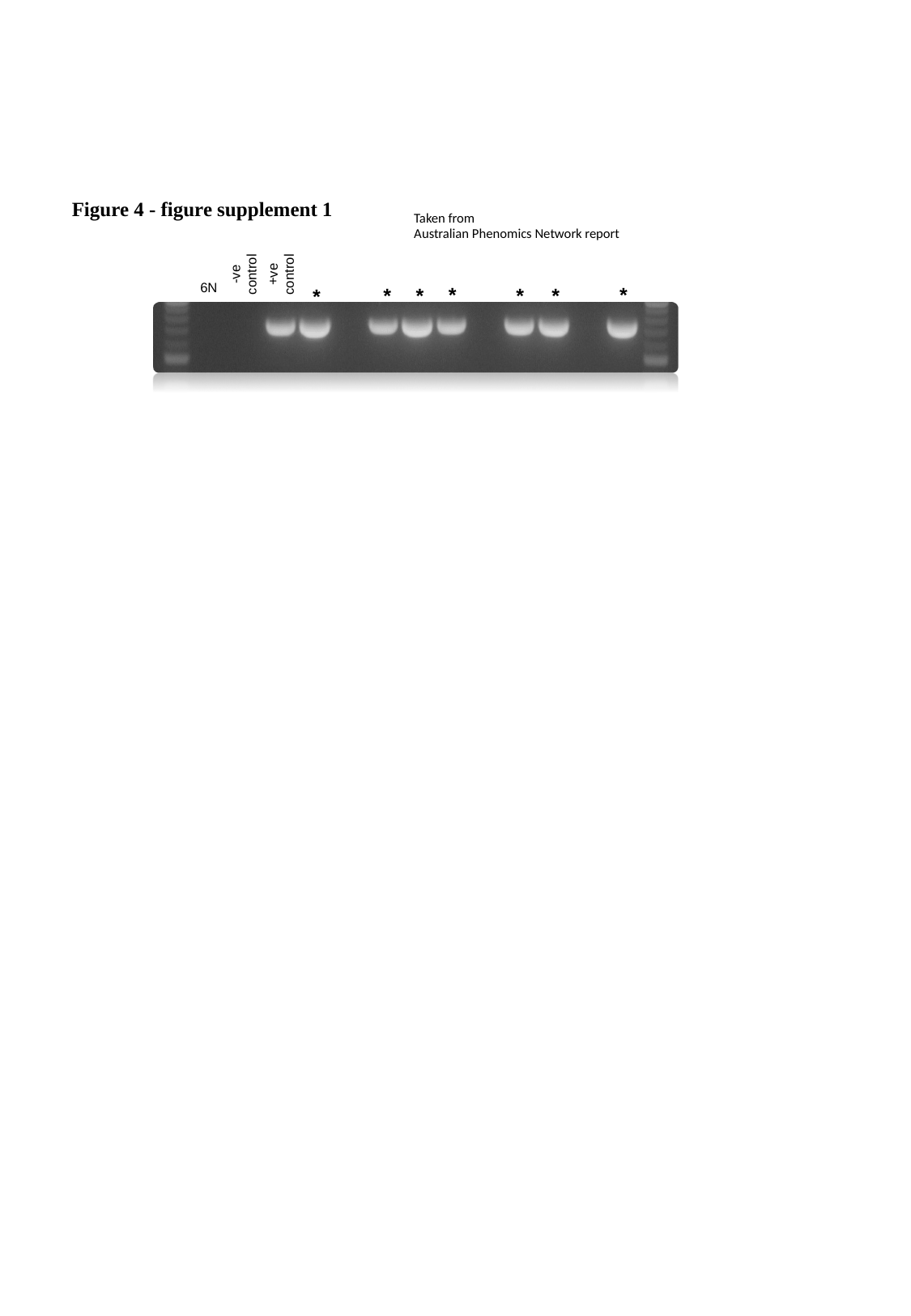

Figure 4 - figure supplement 1
Taken from
Australian Phenomics Network report
-ve
control
+ve
control
6N
*
*
*
*
*
*
*
