# Supplementary material for: Widespread discrepancy in Nnt genotypes and genetic backgrounds complicates granzyme A and other knockout mouse studies
Source: eLife. 2022 Feb 4;11:e70207. doi: 10.7554/eLife.70207 (PMC8816380; doi:10.7554/eLife.70207)
Supplement: Transparent reporting form [file elife-70207-transrepform1.docx]

***eLife’s* transparent reporting form**

We encourage authors to provide detailed information *within their submission* to facilitate the interpretation and replication of experiments. Authors can upload supporting documentation to indicate the use of appropriate reporting guidelines for health-related research (see [EQUATOR Network](http://www.equator-network.org/%20)), life science research (see the [BioSharing Information Resource](https://biosharing.org/)), or the [ARRIVE guidelines](http://www.plosbiology.org/article/info:doi/10.1371/journal.pbio.1000412) for reporting work involving animal research. Where applicable, authors should refer to any relevant reporting standards documents in this form.

If you have any questions, please consult our Journal Policies and/or contact us: [editorial@elifesciences.org](mailto:editorial@elifesciences.org).

**Sample-size estimation**

- You should state whether an appropriate sample size was computed when the study was being designed
- You should state the statistical method of sample size computation and any required assumptions
- If no explicit power analysis was used, you should describe how you decided what sample (replicate) size (number) to use

Please outline where this information can be found within the submission (e.g., sections or figure legends), or explain why this information doesn’t apply to your submission:

There were three pooled RNA replicates per experimental mouse strain. Three replicates per condition are commonly used for RNA-Seq and our pooling strategy (see below) for the replicates was arrived at from experience with similar mouse studies and RNA-Seq. See ‘**RNA-Seq of poly(I:C) injection for GzmAS211A versus 6J mice’**  in Materials and Methods and the legend to **supplementary Figure 9**.

**Replicates**

- You should report how often each experiment was performed
- You should include a definition of biological versus technical replication
- The data obtained should be provided and sufficient information should be provided to indicate the number of independent biological and/or technical replicates
- If you encountered any outliers, you should describe how these were handled
- Criteria for exclusion/inclusion of data should be clearly stated
- High-throughput sequence data should be uploaded before submission, with a private link for reviewers provided (these are available from both GEO and ArrayExpress)

Please outline where this information can be found within the submission (e.g., sections or figure legends), or explain why this information doesn’t apply to your submission:

At least three RNA pooled replicates were generated for each condition for each mouse strain, whereby each pool contained equal amounts of RNA from 4 feet from 4 mice, or 2 spleens from 2 mice. This approach has been used successfully in the past minimizing variation from individual samples .See ‘**RNA-Seq of poly(I:C) injection for GzmAS211A versus 6J mice’**  in Materials and Methods and the Legend to supplementary Figure 9.

Raw sequencing data was uploaded to SRA: Bioproject accession: PRJNA666748

https://dataview.ncbi.nlm.nih.gov/object/PRJNA666748?reviewer=347ek8l18h2jvbt2uvjug29n91

**Statistical reporting**

- Statistical analysis methods should be described and justified
- Raw data should be presented in figures whenever informative to do so (typically when N per group is less than 10)
- For each experiment, you should identify the statistical tests used, exact values of N, definitions of center, methods of multiple test correction, and dispersion and precision measures (e.g., mean, median, SD, SEM, confidence intervals; and, for the major substantive results, a measure of effect size (e.g., Pearson's r, Cohen's d)
- Report exact p-values wherever possible alongside the summary statistics and 95% confidence intervals. These should be reported for all key questions and not only when the p-value is less than 0.05.

Please outline where this information can be found within the submission (e.g., sections or figure legends), or explain why this information doesn’t apply to your submission:

Filtering and significance thresholds for differentially expressed genes are described throughout the Results, Figures and Tables. Upstream analysis of RNA-Seq data (alignment etc) are described in Materials and Methods and Results. Downstream analyses (IPA, GSEA, Cytoscape, K-mer mining etc) tools, methods and statistical attributes and cited throughout the Results, Figures and Tables.

(For large datasets, or papers with a very large number of statistical tests, you may upload a single table file with tests, Ns, etc., with reference to sections in the manuscript.)

**Group allocation**

- Indicate how samples were allocated into experimental groups (in the case of clinical studies, please specify allocation to treatment method); if randomization was used, please also state if restricted randomization was applied
- Indicate if masking was used during group allocation, data collection and/or data analysis

Please outline where this information can be found within the submission (e.g., sections or figure legends), or explain why this information doesn’t apply to your submission:

Five to Six female C57BL/6J mice (6-8 weeks) were randomly allocated to each experimental group / time point. They were were infected with 10^4^ CCID50 CHIKV into each hind foot and treated similarly in all other aspects till sacrifice.

see Materials and Methods and figure legends for description.

**Additional data files (“source data”)**

- We encourage you to upload relevant additional data files, such as numerical data that are represented as a graph in a figure, or as a summary table
- Where provided, these should be in the most useful format, and they can be uploaded as “Source data” files linked to a main figure or table
- Include model definition files including the full list of parameters used
- Include code used for data analysis (e.g., R, MatLab)
- Avoid stating that data files are “available upon request”

Please indicate the figures or tables for which source data files have been provided:

Seven supplementary tables, 2 word documents and 5 excel files with multiple tabs are provided and referenced throughout the manuscript. The Tables provide all the data on which the figures and results are based.
